# IL-34 Regulates Macrophage Polarization and Bone Defect Healing in Aged Mice

**DOI:** 10.3390/ijms27156683

**Published:** 2026-07-27

**Authors:** Wen Pan, Shengao Qin, Zanxu Liu, Jiaqi Wang, Zhaochen Shan

**Affiliations:** 1 Department of Oral and Maxillofacial Surgery, School of Stomatology, Capital Medical University, Beijing 100070, China; dentistpw@163.com (W.P.); liuzanxu1997@163.com (Z.L.); 2 Department of Oral Medicine, School of Stomatology, Capital Medical University, Beijing 100070, China

**Keywords:** Interleukin-34, aging, bone defect healing, macrophage, osteogenesis

## Abstract

Aging is a major risk factor for impaired bone-defect healing, and dysregulation of the immune microenvironment, especially macrophage dysfunction, is closely linked to this process. Interleukin-34 (IL-34) plays a crucial role in macrophage biology; however, its role in aging-related bone-defect healing remains elusive. Herein, young and aged male C57BL/6 mice were utilized to establish a tibial bone defect model. Microcomputed tomography, histological staining, flow cytometry, RNA sequencing and in vitro cell experiments were conducted to explore the role and mechanism of action of IL-34 in aging-impaired bone healing. Aging markedly decreased bone mass, inhibited osteogenic differentiation and impaired bone defect healing in mice. Moreover, aging reduces macrophage numbers at bone defects and suppresses M2 polarization, with the drop likely driven by recruitment, survival or proliferation defects rather than impaired recruitment exclusively. IL-34 was a key differentially expressed gene that was downregulated in aged mice, which was further confirmed at the protein level. Furthermore, aging decreased IL-34 secretion by macrophages and impaired macrophage proliferation and M2 polarisation. Thus, aging impairs tibial bone-defect healing by downregulating IL-34 expression, decreasing macrophage infiltration, and inhibiting M2 polarisation. Furthermore, IL-34 acts as a pro-reparative cytokine that promotes bone healing by regulating macrophage function, suggesting its role as a potential therapeutic target for enhancing age-related bone repair.

## 1. Introduction

Bone defect healing is a highly coordinated physiological process involving sequential stages of inflammation, callus formation and bone remodelling, and relies on the precise interaction of different cell types, cytokines and signalling pathways [1,2]. Among these cellular components, macrophages play a crucial role in the early inflammatory phase and subsequent repair process. They clear necrotic tissue, secrete pro-reparative cytokines, and facilitate osteoprogenitor cell recruitment and differentiation [3,4]. Macrophages show phenotypic plasticity, mainly polarising into pro-inflammatory (M1) or pro-reparative (M2) subtypes. CD206^+^ M2-like macrophages play a critical role in promoting tissue repair and regeneration. They secrete anti-inflammatory and osteogenic cytokines, clear cellular debris, and enhance the recruitment and differentiation of mesenchymal stem cells, thereby facilitating extracellular matrix remodeling and bone defect healing [3].

Aging is a well-recognised risk factor for impaired bone repair, and is characterised by delayed callus formation, decreased osteogenic differentiation and poor bone quality [5,6]. With an aging global population, the incidence of age-related bone defects and delayed union continues to increase, posing considerable clinical and socioeconomic burdens. Aging changes the immune microenvironment at the bone defect site, including impaired macrophage recruitment and abnormal polarisation, which contribute to compromised bone healing capacity in aged individuals [7,8]. However, the molecular mechanisms underlying the aging-induced dysregulation of macrophage function and bone repair remain elusive.

Interleukin-34 (IL-34), a cytokine first identified in 2008, shares functional similarities with the macrophage colony-stimulating factor (M-CSF) and binds to the same receptor (c-Fms) to regulate macrophage survival, proliferation and differentiation [9]. IL-34 is widely expressed in different tissues, including bone, and has been implicated in the regulation of inflammatory responses, tissue repair and bone metabolism [10,11]. For example, IL-34 promotes macrophage recruitment and M2 polarization in tissue injury models, and its dysregulation is linked to different inflammatory and degenerative diseases [12,13,14]. However, its role in aging-related bone defect healing and its regulatory effect on macrophage polarisation remains unclear.

Herein, we established a mouse model of tibial bone defect to assess the effects of aging on bone healing. We combined micro-computed tomography (micro-CT), histological staining, flow cytometry, RNA sequencing (RNA-seq) and in vitro cell culture experiments to explore the molecular mechanisms underlying aging-impaired bone repair. IL-34 was found to be a primary differentially expressed gene regulated by aging and further validated its role in promoting macrophage proliferation and pro-reparative M2 polarisation. This study aimed to clarify IL-34’s role in aging-related bone defect healing and provides a potential therapeutic target for enhancing bone repair in the elderly.

## 2. Results

### 2.1. Aging Impairs Tibial Bone Defect Healing In Vivo

**Figure 1:**
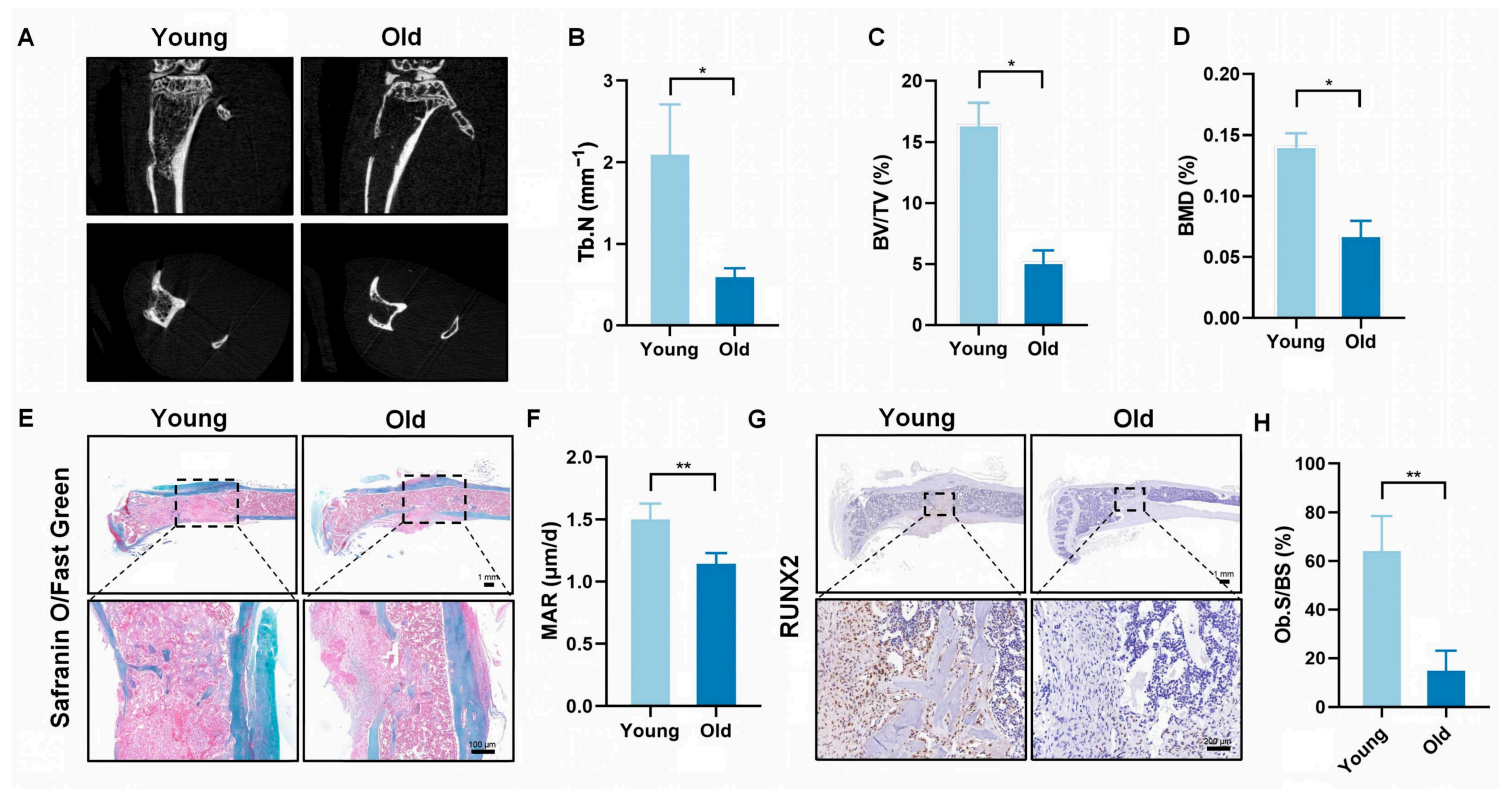
Aging impairs tibial bone defect healing in mice. (**A**) Representative micro-CT 3D reconstruction images of tibial bone defects collected at three weeks postsurgery. (**B**–**D**) Quantitative analysis of Tb.N, BV/TV and BMD derived from micro-CT data. (**E**) Safranin O/Fast Green staining of tibial bone defect sections at one week postsurgery. Low-magnification panoramic images (scale bar = 1 mm) show the full range of bone defects; high-magnification magnified views (scale bar = 100 μm) exhibit local tissue microstructure. (**F**) Quantification of mineral apposition rate (MAR). (**G**) RUNX2 immunohistochemical staining of bone defect tissues at one week postsurgery. Low-magnification overviews (scale bar = 1 mm) display complete defect regions; magnified insets (scale bar = 200 μm) show RUNX2-positive cells. (**H**) Histomorphometric analysis of Ob.S/BS (osteoblast surface/bone surface). All quantitative data are presented as mean ± standard deviation. *n* = 9 per group. * *p* < 0.05, ** *p* < 0.01.

Micro-computed tomography (micro-CT) and histological analyses were conducted at 3 weeks following tibial bone defect surgery to assess the effect of aging on bone repair. Sampling time points were defined based on preliminary experiments. Histology at week 1 captures early osteogenic initiation, while quantifiable mineralized callus for micro-CT only develops by week 3. Three weeks postoperatively, micro-CT imaging (Figure 1A) showed that young mice exhibited abundant callus formation with dense and continuous trabecular structures within the defect region. In contrast, aged mice displayed substantially reduced bone mass, sparse callus and discontinuous cortical bone. Quantitative analysis revealed significantly higher Tb.N, BV/TV and BMD in young mice compared with aged mice (Figure 1B–D).

One week postoperatively, Safranin O/Fast Green staining (Figure 1E) demonstrated increased newly formed bone tissue in young mice, while aged mice predominantly possessed fibrous tissue accompanied by delayed osteogenesis. The low-magnification panoramic images confirmed consistent positioning of tibial bone defects across all animals. Mineral apposition rate (MAR) was significantly lower in aged mice (Figure 1F).

Immunohistochemical staining for RUNX2, a master transcription factor governing osteogenic differentiation, revealed markedly more extensive positive signals within defects of young animals (Figure 1G). Histomorphometric quantification further verified that Ob.S/BS (osteoblast surface/bone surface) was significantly elevated in young mice versus aged mice (Figure 1H).

Collectively, these data indicate aging suppresses early osteogenic differentiation and impairs bone defect regeneration in aged mice.

### 2.2. Aging Decreases Macrophage Infiltration and Pro-Healing Polarization at the Early Stage of Bone Defect

**Figure 2:**
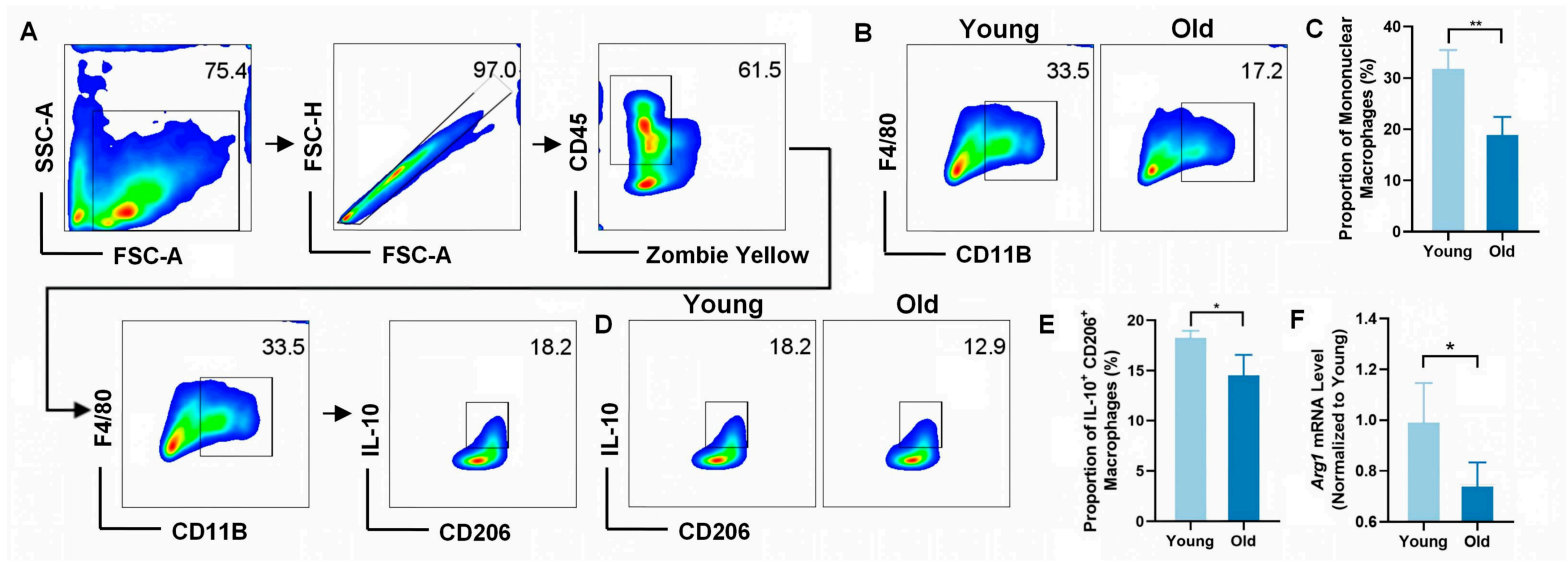
Aging decreases macrophage infiltration and pro-healing polarization in the bone marrow at the early bone repair stage. (**A**) Flow cytometric analysis of the bone marrow cells isolated from the bone defect area at one week post-surgery. (**B**,**C**) Representative flow cytometry plots and quantitative analysis of the F4/80^+^CD11b^+^ total macrophages. (**D**,**E**) Representative flow cytometric plots and quantitative analysis of the CD11b^+^CD206^+^ M2 reparative macrophages with IL-10 expression. (**F**) qPCR quantification of M2 marker *Arg1* mRNA. Data represent mean ± standard deviation from three independent experiments. * *p* < 0.05, ** *p* < 0.01.

Flow cytometry was performed on bone marrow cells isolated from the defect region one week after surgery to examine the immune microenvironment during bone repair (Figure 2A).

The proportion of the total macrophages (Figure 2B,C, F4/80^+^CD11b^+^) was 33.5% in young mice, which was markedly higher than that in aged mice (17.2%). Additionally, the percentage of CD206^+^ M2 reparative macrophages with IL-10 anti-inflammatory function (Figure 2D,E, CD206^+^IL-10^+^ double-positive cells) was 18.2% in young mice, which was substantially higher than 12.9% in aged mice. We further detected mRNA levels of the M2 marker Arg-1 (Figure 2F), which was significantly lower in aged macrophages, further supporting impaired reparative polarization. Thus, aging reduces the total number of macrophages within bone defect tissue and inhibits their polarization toward the reparative M2 phenotype.

### 2.3. Transcriptomic Analysis Identifies IL-34 as a Key Differentially Expressed Gene Regulated by Aging

**Figure 3:**
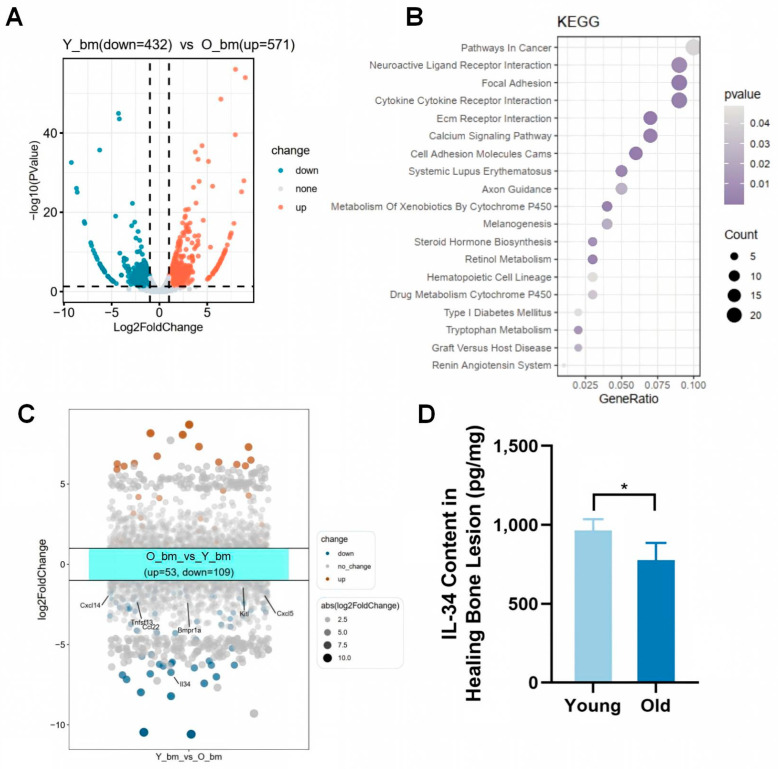
Transcriptomic screening identifies IL-34 as the most crucial differentially expressed gene (DEG) within the cytokine–cytokine receptor interaction pathway in aged bone defects. (**A**) Volcano plot of DEGs between young and aged mouse bone defect tissues. Vertical dashed lines: the cut-off threshold of |Log_2_FoldChange| = 1, used to screen significantly upregulated and downregulated genes. (**B**) Kyoto Encyclopedia of Genes and Genomes enrichment analysis revealing the top enriched pathways; cytokine–cytokine receptor interaction was the most significantly enriched pathway. (**C**) Expression heatmap of the DEGs in the cytokine–cytokine receptor interaction pathway. (**D**) Enzyme-linked immunosorbent assay quantification of the IL-34 protein levels in the bone defect tissues. *n* = 6 per group. Values are expressed as mean ± standard deviation. * *p* < 0.05.

RNA sequencing (RNA-seq) was conducted on bone-defect tissues from young and aged mice to investigate the molecular mechanisms underlying aging-impaired bone healing. Volcano plot analysis identified several differentially expressed genes (DEGs) between the two groups (Figure 3A). Kyoto Encyclopedia of Genes and Genomes (KEGG) enrichment analysis showed that the top enriched pathways included cytokine cytokine receptor interaction, and IL-34 was the most significantly downregulated gene within this cytokine–cytokine receptor interaction pathway in aged mice (Figure 3B). Further visualisation of the DEGs in this pathway revealed that IL-34 was the most significantly downregulated gene in aged mice (Figure 3C). Enzyme-linked immunosorbent assay (ELISA) confirmed that the protein level of IL-34 in bone defect tissues was substantially higher in young mice than in aged mice (Figure 3D), which is consistent with the transcriptomic findings. Notably, IL-34 exhibited a much larger fold-change reduction than other macrophage-regulating cytokines altered with aging in this pathway.

### 2.4. Aging Reduces IL-34 Secretion, Proliferation, and M2 Polarization of Macrophages

**Figure 4:**
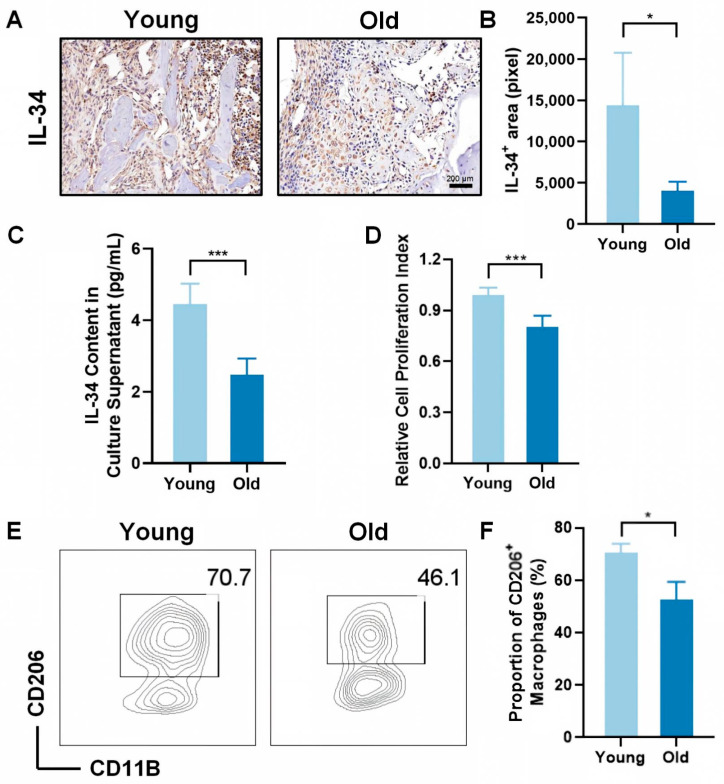
Aging reduces IL-34 secretion, proliferation, and M2 polarization of macrophages. (**A**,**B**) Immunohistochemical staining and quantitative analysis of IL-34 expression in bone defect tissues. Magnified insets (scale bar = 200 μm). (**C**) Enzyme-linked immunosorbent assay (ELISA) measurement of IL-34 secretion in the supernatants of cultured bone marrow-derived macrophages (1 × 10^6^ cells/well in 6-well plates). (**D**) CCK-8 assay revealing the proliferative capacity of young and aged macrophages. (**E**,**F**) Flow cytometric analysis and quantification of the CD11b^+^CD206^+^ reparative macrophages. Data represent mean ± standard deviation from three independent experiments. * *p* < 0.05, *** *p* < 0.001.

Immunohistochemical staining confirmed that the IL-34-positive area was substantially larger in the bone defect region of young mice than in that of aged mice (Figure 4A,B). Bone marrow-derived macrophages were cultured in 6-well plates at a density of 1 × 10^6^ cells per well. ELISA of the culture supernatants showed that the macrophages from young mice secreted significantly more IL-34 than those from aged mice (Figure 4C). CCK-8 proliferation assay showed that aged macrophages exhibited a substantially lower proliferation rate than young macrophages (Figure 4D). Flow cytometric analysis further revealed that the proportion of CD11b^+^CD206^+^ reparative macrophages was 70.7% in young mice, which was significantly higher than 46.1% in aged mice (Figure 4E,F). Thus, aging decreases IL-34 expression and impairs macrophage proliferation and M2 polarisation.

### 2.5. IL-34 Remodels the Repair-Associated Transcriptional Profile of Macrophages

**Figure 5:**
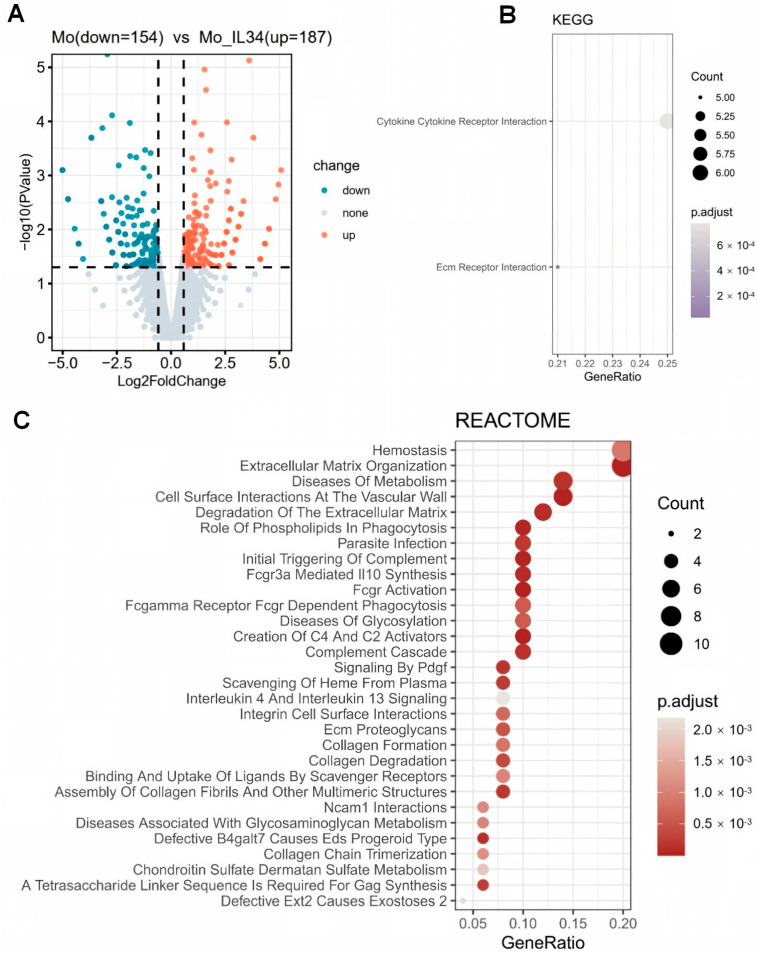
IL-34 remodels the transcriptomic profile of macrophages towards a pro-repair phenotype. Macrophages were treated with recombinant IL-34 for three days and subsequently subjected to RNA-seq. (**A**) Volcano plot of the differentially expressed genes (DEGs) between the control and IL-34-treated macrophages. (**B**) Kyoto Encyclopedia of Genes and Genomes enrichment analysis of the DEGs. (**C**) REACTOME enrichment analysis of the DEGs. Enriched pathways associated with tissue repair are highlighted.

Cultured macrophages were treated with recombinant IL-34 for 3 days, followed by RNA-seq analysis to determine the direct effect of IL-34 on macrophages (Figure 5). DEG analysis revealed global transcriptional alterations induced by IL-34 (Figure 5A). KEGG and REACTOME enrichment analyses showed that DEGs were markedly enriched in pathways associate with tissue repair, including cytokine cytokine receptor interactions and Ecm receptor interactions (Figure 5B,C). Thus, IL-34 directly programs macrophages towards a prorepair functional phenotype.

### 2.6. IL-34 Promotes Macrophage Proliferation and Reparative M2 Polarization In Vitro

**Figure 6:**
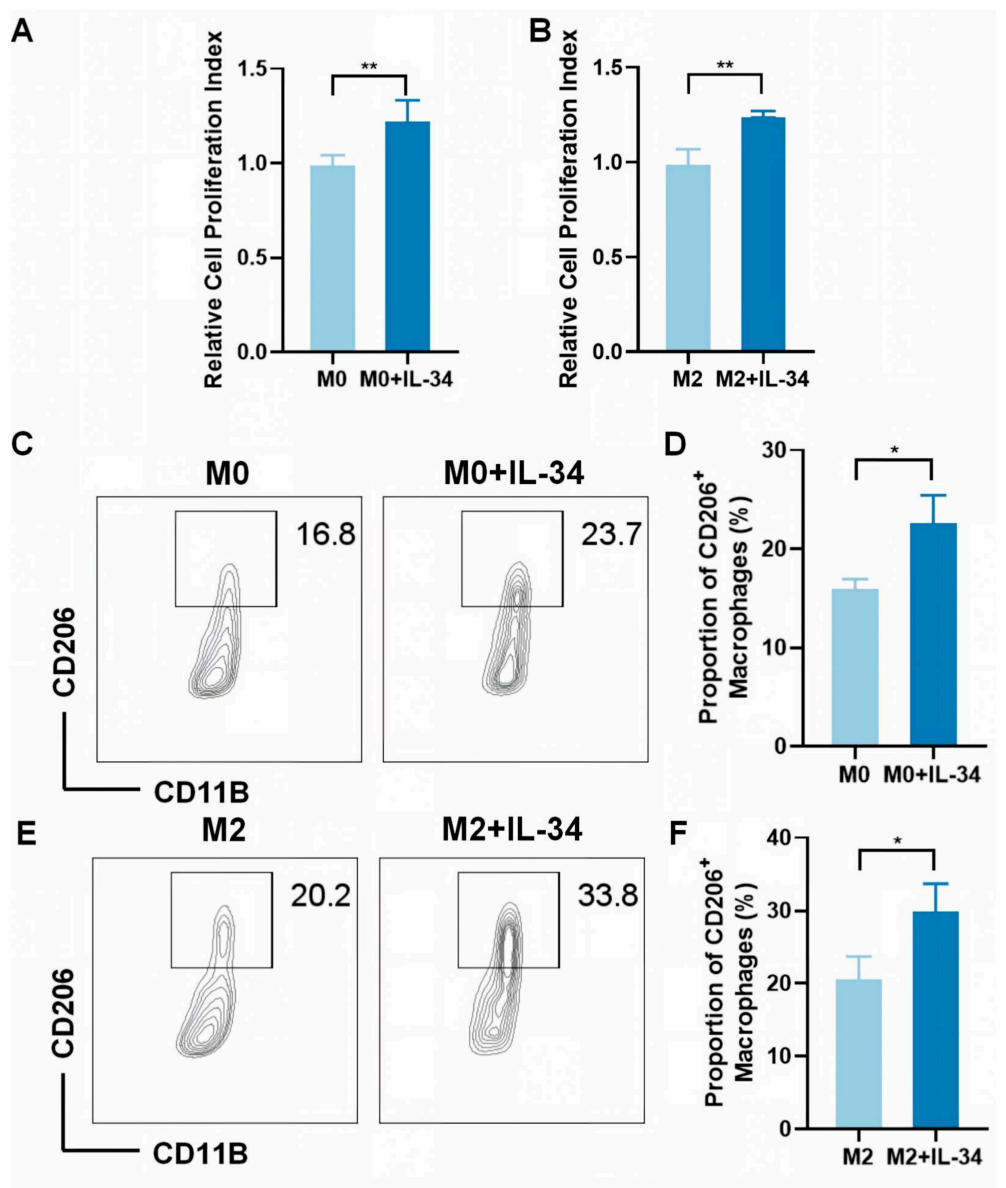
IL-34 promotes macrophage proliferation and reparative M2 polarization in vitro. (**A**,**B**) CCK-8 proliferation assay of the M0 and M2 macrophages following IL-34 treatment. (**C**–**F**) Flow cytometric analysis and quantification of the CD206^+^ reparative macrophages in the M0 and M2 macrophage populations following IL-34 treatment. Data represent mean ± standard deviation from three independent experiments. * *p* < 0.05, ** *p* < 0.01.

The regulatory effects of IL-34 on macrophage proliferation and polarisation were validated under in vitro conditions. CCK-8 proliferation assays revealed that IL-34 treatment markedly enhanced M0 and M2 macrophage proliferation (Figure 6A,B). Flow cytometry analysis showed that IL-34 substantially increased the proportion of CD206^+^ reparative macrophages from 16.8% to 23.7% in M0 macrophages and from 20.2% to 33.8% in M2 macrophages (Figure 6C–F). Collectively, these results confirm that IL-34 directly promotes macrophage proliferation and drives their polarisation towards a reparative M2 phenotype.

### 2.7. Local IL-34 Administration Rescues Impaired Aged Bone Defect Healing

**Figure 7:**
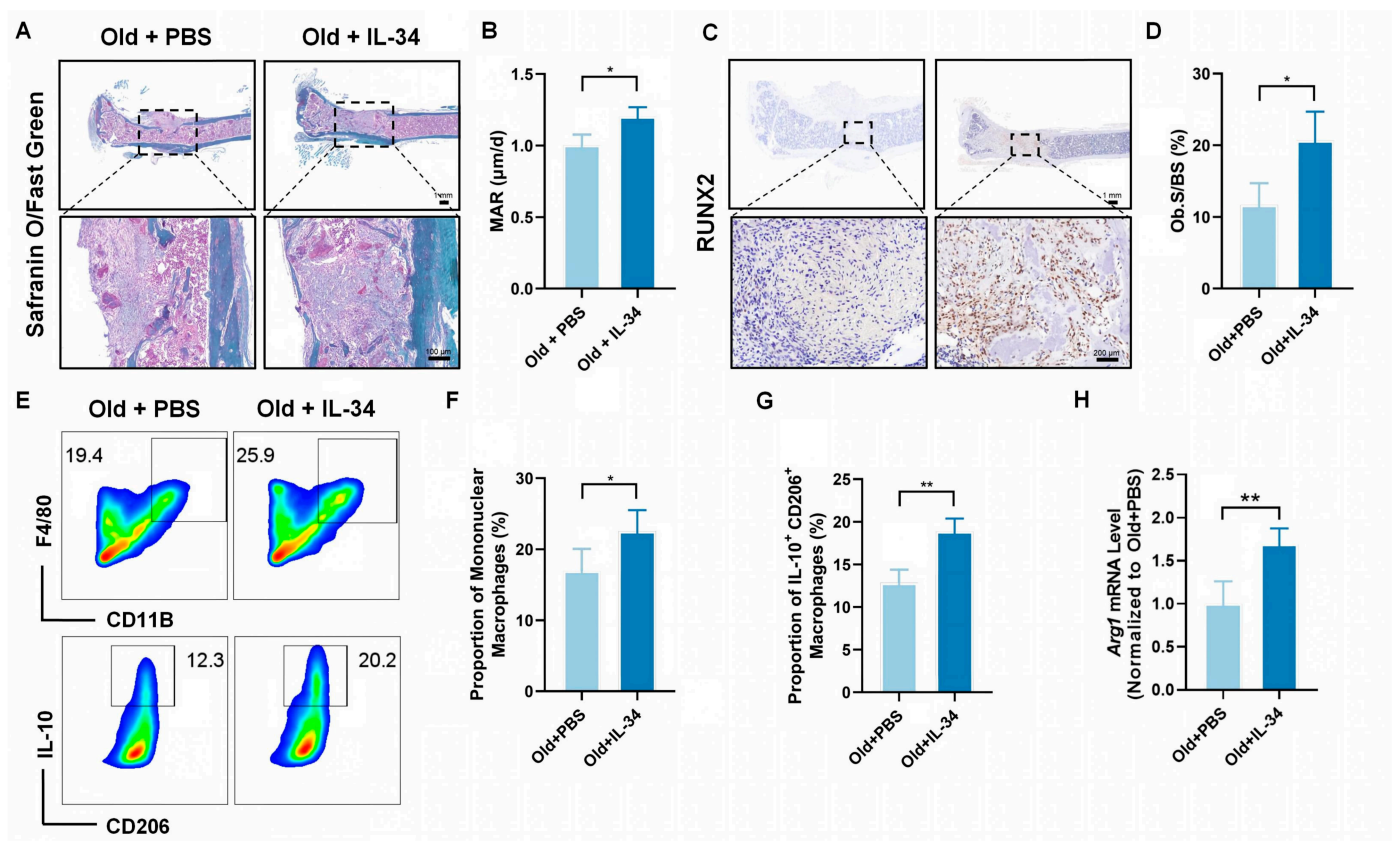
Local recombinant IL-34 injection rescues impaired early bone defect healing in aged mice. (**A**) Safranin O/Fast Green staining of tibial bone defects in Old + PBS and Old + IL-34 groups at 1 week post-surgery. Low-magnification panoramic images (scale bar = 1 mm) show complete defect regions; high-magnification magnified views (scale bar = 100 μm) display local tissue microstructure. (**B**) Quantification of mineral apposition rate (MAR). (**C**) RUNX2 immunohistochemical staining of bone defect tissues. Low-magnification overviews (scale bar = 1 mm) show full defect sites; magnified insets (scale bar = 200 μm) visualize RUNX2-positive osteoblasts. (**D**) Histomorphometric analysis of Ob.S/BS (osteoblast surface/bone surface). (**E**–**G**) Flow cytometry analysis of bone marrow macrophages: (**E**) Representative gating plots for F4/80^+^CD11B^+^ mononuclear macrophages and IL-10^+^CD206^+^ reparative macrophages; (**F**) Statistical proportion of total mononuclear macrophages; (**G**) Percentage of IL-10^+^CD206^+^ M2 macrophages. (**H**) Relative mRNA expression of M2 macrophage marker *Arg1*, normalized to the Old + PBS group. All quantitative data are presented as mean ± standard deviation. *n* = 6 per group. * *p* < 0.05, ** *p* < 0.01.

To directly verify IL-34’s critical role in aged bone repair, we performed local recombinant IL-34 injection into tibial bone defects of aged mice (PBS as vehicle control). Safranin O/Fast Green staining (Figure 7A) showed IL-34 treatment significantly increased newly formed bone tissue and reduced fibrous tissue in aged defects, with obvious restoration of cortical bone continuity in the IL-34 rescue group. Corresponding histomorphometric quantification of MAR and Ob.S/BS further validated improved osteogenic activity after IL-34 intervention (Figure 7B,D). RUNX2 IHC staining (Figure 7C) further confirmed exogenous IL-34 markedly upregulated osteogenic RUNX2 expression in aged bone microenvironment. To characterize the upstream immune regulatory mechanism, we conducted flow cytometry analysis on bone marrow macrophages. IL-34 injection elevated the proportion of total mononuclear macrophages and reparative IL-10^+^CD206^+^ macrophages (Figure 7E–G). Consistently, qPCR results showed the M2 macrophage marker Arg1 was significantly upregulated after IL-34 treatment (Figure 7H).

These in vivo rescue data directly confirm IL-34 rescues age-related impaired bone healing, strongly supporting our core conclusion. Early osteogenic activation acts as the prerequisite for late-stage bone maturation. The restored pro-regenerative signals induced by IL-34 at this early 1-week repair window can sustainably drive subsequent full bone remodeling and mature bone formation.

## 3. Discussion

Consistent with previous studies, our results show that aging markedly impairs the healing of tibial bone defects in mice. At three weeks postsurgery, micro-CT analysis showed that aged mice had substantially reduced bone mass, sparse callus formation and poor cortical continuity compared to young mice. This was confirmed by the markedly lower bone volume/tissue volume (BV/TV) ratio in aged mice. Histologically, young mice revealed more newly formed bone tissue one week after surgery, whereas aged mice predominantly showed fibrous and cartilaginous tissue, indicating delayed osteogenesis. RUNX2, a primary transcription factor that drives osteogenic differentiation and bone formation [15], was expressed at significantly lower levels in bone defect tissues of aged mice, further confirming that aging suppresses early osteogenic differentiation. These results align with the well-documented phenomenon that aging compromises bone regenerative capacity, and that delayed osteogenesis in aged mice may be linked to decrease activity of osteoprogenitor cells, impaired angiogenesis and dysregulation of the immune microenvironment [6,16]. Our study focused on the immune microenvironment, especially macrophage function, and provided new insights into the mechanisms underlying aging-induced bone healing impairment.

Macrophages are necessary for the early phase of bone healing because they initiate an inflammatory response, clear debris, and secrete cytokines that promote osteogenesis and angiogenesis [3,17]. Our flow cytometry findings revealed that aging substantially reduced the total macrophage recruitment (F4/80^+^CD11b^+^) to the bone defect site, with the proportion of total macrophages being nearly half in aged mice compared to that in young mice. Additionally, aging inhibited macrophage polarisation towards the pro-reparative M2 phenotype, as evidenced by a markedly lower proportion of CD11b^+^IL-10^+^ reparative macrophages in aged mice. This change may contribute to delayed bone healing by impairing necrotic tissue clearance and proreparative cytokine secretion. Moreover, restoring M2 macrophage polarisation can enhance bone healing in aged animals [18]. This underscores the criticality of the macrophage phenotype balance in aging-related bone repair and confirming that macrophage dysfunction is a primary feature of aging-impaired bone healing.

We conducted RNA sequencing of bone defect tissues from young and aged mice to identify the molecular mechanisms underlying aging-impaired bone healing. KEGG enrichment analysis showed that the cytokine–cytokine receptor interaction pathway was substantially enriched. This indicated the crucial role of dysregulated cytokine signalling. Among the DEGs in this pathway, IL-34 was the most substantially downregulated gene in aged mice, which was further confirmed at the protein level using ELISA. IL-34 downregulation may contribute to impaired macrophage function as a key regulator of macrophage biology. IL-34 is involved in the regulation of macrophage survival and polarisation, and its deficiency influences tissue repair [9,10,12,13]. Therefore, aging-induced IL-34 downregulation may be a key molecular event driving the dysregulation of the immune microenvironment and impaired bone healing in aged mice.

Notably, among all cytokine genes with age-dependent expression shifts in bone defect tissue, IL-34 exhibited the most striking downregulation in aged mice with a far larger fold change than other macrophage-modulating cytokines. Distinct from other cytokines with mild age-related expression fluctuations, local supplementation of recombinant IL-34 alone was sufficient to rescue age-induced defects in macrophage proliferation and M2 polarization, and further restore impaired osteogenic capacity. Combined with our in vitro macrophage stimulation results, these lines of evidence collectively support that IL-34 acts as a central age-associated cytokine governing bone repair immune microenvironment, rather than merely one of many moderately altered inflammatory mediators.

Immunohistochemical staining and in vitro macrophage culture experiments revealed that IL-34 expression was markedly lower in the bone defect tissues of aged mice than in those of young mice. Under in vitro conditions, macrophages from aged mice secreted markedly less IL-34, showed a lower proliferation rate, and had a significantly decreased proportion of CD11b^+^CD206^+^ reparative macrophages (a marker of M2 polarisation) [19]. Thus, aging decreases IL-34 expression in bone-defect tissues and impairs the ability of macrophages to secrete IL-34, proliferate and polarise into the M2 phenotype. Reduced IL-34 secretion by aged macrophages may form a vicious cycle, further inhibiting macrophage function and exacerbating impaired bone healing. This highlights IL-34 as a potential link between aging, macrophage dysfunction and impaired bone repair.

Although no studies have directly confirmed the role of IL-34 in age-related bone healing, previous reports have shown that IL-34 regulates M2 macrophage polarization and alleviates inflammatory damage in aging-related skin and rheumatoid arthritis diseases [10,12]. Our study verified that IL-34 promotes M2 macrophage polarization in the bone defect microenvironment. Given that M2 macrophages are essential for osteogenic differentiation and bone tissue repair [3], we conclude that downregulation of IL-34 during aging contributes to impaired bone healing, while IL-34-mediated M2 polarization effectively promotes bone repair.

Treatment of cultured macrophages with recombinant IL-34 and RNA sequencing revealed that IL-34 induced global transcriptional alterations in macrophages, with DEGs markedly enriched in tissue repair-related pathways, including cytokine–cytokine receptor interactions and extracellular matrix remodelling, which are crucial for macrophage-mediated tissue repair [20]. In vitro functional experiments confirmed that IL-34 significantly promoted the proliferation of M0 and M2 macrophages and increased the proportion of CD206^+^ reparative macrophages, consistent with the results of previous studies that showed that IL-34 improves M2 polarisation and tissue repair [10,21]. Thus, IL-34 acts as a proreparative cytokine that promotes bone healing by remodelling the macrophage function.

The present study identified IL-34 as a key regulator of aging-impaired bone defect healing as it promotes macrophage proliferation and M2 polarisation, which has crucial clinical implications. IL-34 is a potential therapeutic target for improving bone repair in the elderly. Furthermore, exogenous administration or strategies to enhance its endogenous expression can restore macrophage function and promote bone healing in the elderly.

To further validate the essential role of IL-34 in aged bone repair, we performed in vivo local IL-34 rescue experiments in aged mice. Results showed that local injection of recombinant IL-34 significantly increased new bone formation and upregulated RUNX2 expression in aged bone defects, confirming that IL-34 supplementation effectively rescues age-related impaired bone healing. These in vivo findings provide direct evidence supporting our conclusion that IL-34 critically regulates bone defect healing in aged mice.

However, this study has certain limitations. First, we have supplemented in vivo IL-34 rescue experiments to verify the critical role of IL-34 in aged bone defect healing, but more definitive evidence may require further gene knockout or transgenic models in future studies. Second, we only evaluated the effect of IL-34 on macrophages, and its direct effects on osteoblasts and osteoclasts remain to be explored. Third, this study was based on a mouse tibial bone defect model, and the translational potential of our findings needs to be verified in clinical studies. Finally, the downstream signaling pathways through which IL-34 regulates macrophage proliferation and polarization were not explored, which is worthy of further investigation.

In conclusion, our findings demonstrate that aging hinders tibial bone repair by lowering IL-34 levels, reducing macrophage abundance and suppressing reparative M2 polarization. As a pro-repair cytokine, IL-34 directly boosts macrophage proliferation and M2 polarization to ameliorate aged bone regeneration. Published studies confirm IL-34 signals primarily via macrophage CSF1R; targeted blocking assays will be implemented in our follow-up research to dissect this precise regulatory cascade. This work delivers preliminary mechanistic insights and identifies IL-34 as a candidate therapeutic target for age-related bone defects.

## 4. Materials and Methods

### 4.1. Animals

Young (2–3 months old) and aged (18–20 months old) male C57BL/6 mice were purchased from the Jackson laboratory. All mice were housed under specific pathogen-free (SPF) conditions with a 12 h light/dark cycle, free access to food and water. Mice were randomly allocated to experimental groups using a computer-generated random number table prior to surgery. All outcome assessments were performed by investigators blinded to group allocation to eliminate observer bias. Based on our preliminary experimental data, established protocols from published bone defect healing studies [22,23], and strict adherence to the 3R ethical principles to minimize animal usage, a sample size of *n* = 8 mice per group was determined for all experiments described in Section 4.2, Section 4.3, Section 4.4, Section 4.5, Section 4.6, Section 4.7, Section 4.8 and Section 4.9. This sample size is widely accepted in the bone regeneration field and provides sufficient statistical power (power > 0.8, α = 0.05) to detect significant differences between groups. All animal experiments were approved by the Animal Care and Use Committee of Capital Medical University (Approval No.: AEEI-2024-281) and conducted in accordance with the Guide for the Care and Use of Laboratory Animals.

### 4.2. Tibial Bone Defect Model Establishment

Mice were anesthetized with isoflurane (2–3% for induction, 1–1.5% for maintenance). The right hindlimb was shaved and disinfected with 75% ethanol. A 1 cm longitudinal incision was made along the lateral side of the tibia, and the muscle was bluntly separated to expose the tibial diaphysis. A bone defect with a diameter of 1 mm and a depth of 1 mm was created in the mid-diaphysis of the tibia using a micro-drill (Shanghai Surgical Instrument Co., Ltd., Shanghai, China) at a constant speed of 1000 rpm, with continuous sterile saline irrigation to prevent thermal injury to the surrounding bone tissue [16]. For the in vivo IL-34 rescue experiment, aged mice were randomly divided into two groups immediately after defect establishment: Old + PBS group: received a single local injection of 20 μL PBS into the bone defect cavity; and Old + IL-34 group: received a single local injection of 20 μL recombinant mouse IL-34 (0.01 ng/mL) into the bone defect cavity [24]. The incision was sutured layer by layer with 4-0 absorbable sutures. All mice were euthanized at 1 week (7 days) after surgery for subsequent histological and immunohistochemical analyses.

### 4.3. Micro-CT Analysis

At 3 weeks post-surgery, mice were sacrificed by cervical dislocation, and the right tibia was harvested and fixed in 4% paraformaldehyde for 24 h. Micro-CT scanning was performed using a SkyScan 1276 micro-CT system (Bruker, Kontich, Belgium) with the following parameters: voltage = 50 kV, current = 500 μA, pixel size = 9 μm, rotation step = 0.5°, aluminum filter = 0.5 mm, exposure time = 300 ms. The region of interest (ROI) was set as the area surrounding the bone defect (500 μm above and below the defect). Bone volume/tissue volume (BV/TV) was analyzed using CTAn software (v.1.21.2.0, Bruker, Kontich, Belgium).

### 4.4. Histological Staining and Immunohistochemistry

Tibial samples were decalcified in 10% EDTA (pH 7.4) for 4 weeks, embedded in paraffin, and sectioned into 5 μm thick slices. Safranin-o-carmine staining to observe the osteogenic structure. For immunohistochemical staining, sections were deparaffinized, rehydrated, and antigen-retrieved in citrate buffer (pH 6.0) for 15 min. After blocking with 5% BSA for 1 h at room temperature, sections were incubated with primary antibody against IL-34 (1:500, Invitrogen, Carlsbad, CA, USA) or RUNX2 (1:200, Invitrogen, Carlsbad, CA, USA) overnight at 4 °C. After washing with PBS, sections were incubated with horseradish peroxidase (HRP)-conjugated secondary antibody (1:500, Invitrogen, Carlsbad, CA, USA) for 1 h at room temperature. The staining was visualized using 3,3′-diaminobenzidine (DAB) substrate, and hematoxylin was used for counterstaining of cell nuclei. Sections were then dehydrated, cleared, and mounted with neutral balsam. Antibody specificity was validated by the manufacturer through Western blotting, immunoprecipitation, and immunohistochemistry, with specificity confirmed in peer-reviewed literature [25,26]. For additional confirmation of antibody specificity, IHC assays were carried out using paraffin sections of IL-34 KO and RUNX2 KO mice obtained from Cyagen Biosciences (Suzhou, China). No target-specific staining was seen in knockout specimens (Supplementary Figure S1).

Images were captured using a light microscope (Olympus, Wetzlar, Germany). A standardized ROI covering the entire bone defect area was analyzed. For RUNX2, the percentage of DAB-positive RUNX2^+^ nuclei relative to total hematoxylin-stained nuclei was calculated to reflect osteogenic activity. For IL-34, the percentage of DAB-positive IL-34-expressing cells (nuclear/cytoplasmic staining) relative to total nuclei was calculated. All counting was performed in a blinded manner using Image-Pro Plus software (version 6.0, Media Cybernetics, Rockville, MD, USA).

### 4.5. Flow Cytometry Analysis

At 1 week post-surgery, bone defect tissues were harvested and minced into small pieces, then digested with collagenase type I (1 mg/mL, Sigma-Aldrich, St. Louis, MO, USA) and DNase I (100 μg/mL, Sigma-Aldrich) at 37 °C for 30 min. Single-cell suspensions were prepared by passing through a 70 μm cell strainer. After washing with PBS, cells were stained with fluorochrome-conjugated antibodies: CD11b (1:100), F4/80 (1:100), IL-10 (1:100), and CD206 (1:100) (all from BD Biosciences, Milpitas, CA, USA) for 30 min at 4 °C in the dark. Isotype controls were used to exclude non-specific staining. The gating strategy was as follows: (1) Gating on single cells via FSC-A vs. FSC-H; (2) Gating on live cells via viability staining; (3) Gating on total macrophages via F4/80^+^CD11b^+^; (4) Sub-gating on M2 macrophages via CD206^+^. CD206 is a well-recognized surface marker for pro-reparative M2 macrophages in bone regeneration research, and IL-10 was used as a supplementary functional marker to confirm the anti-inflammatory phenotype [27]. Flow cytometry was performed using a BD FACSymphony flow cytometer (BD Biosciences, USA), and data were analyzed using FlowJo software (version 10.8.1).

### 4.6. RNA Sequencing and Bioinformatics Analysis

Total RNA was extracted from bone defect tissues using Trizol reagent (Invitrogen) according to the manufacturer’s instructions. RNA quality was assessed using a NanoDrop 2000 spectrophotometer (Thermo Fisher Scientific, Waltham, MA, USA) and agarose gel electrophoresis. RNA sequencing was performed by Annoroad Gene Technology (Beijing, China) using an Illumina NovaSeq 6000 platform (Illumina, Inc., San Diego, CA, USA), with *n* = 3 independent biological replicates per group. Differentially expressed genes (DEGs) were identified with |log2FC| > 1 and adjusted *p*-value < 0.05. Kyoto Encyclopedia of Genes and Genomes (KEGG) enrichment analysis was performed using ClusterProfiler R package (version 4.10.0) to identify significantly enriched pathways.

### 4.7. Real-Time RT-PCR

Total RNA was extracted from homogenized mouse lungs or cultured cells using the RNeasy mini kit (Qiagen, Germantown, MD, USA), and then reverse-transcribed to cDNA using the PrimeScript RT Reagent Kit (RR037A, Takara Bio, Tokyo, Japan). The PCR mixture comprised 10 μL SYBR Green Master Mix, 0.5 μM forward and reverse primers, and 2 μL cDNA sample. After normalization of target gene expression, data were quantified using the 2^−ΔΔCt^ method. We normalized mRNA expressions for *Gapdh*.

### 4.8. ELISA

The concentration of IL-34 in bone defect tissues and macrophage culture supernatants was detected using a mouse IL-34 ELISA kit (R&D Systems, Minneapolis, MN, USA) according to the manufacturer’s instructions. Briefly, samples were added to 96-well plates coated with anti-IL-34 antibody, incubated at room temperature for 2 h, and then incubated with detection antibody for 1 h. After washing, streptavidin-HRP was added, and the plates were incubated for 30 min. TMB substrate was added, and the reaction was stopped with stop solution. The absorbance at 450 nm was measured using a microplate reader.

### 4.9. Macrophage Isolation and Culture

Bone marrow was harvested, and red blood cells were then eliminated using a specialized lysis reagent (BioLegend, San Diego, CA, USA). To isolate monocytes, cells positive for Gr1 (a granulocyte marker), B220 (a B-cell marker), and Ter119 (an erythroid lineage marker) were depleted through magnetic column-based separation. The purified monocytes were then cultured in RPMI 1640 medium supplemented with fetal bovine serum (FBS) and macrophage colony-stimulating factor (M-CSF) to allow spontaneous differentiation into M0 macrophages. Following 5 days of culture, the adherent M0 macrophages were harvested and seeded into multiwell plates and allowed to adhere overnight before stimulation. M0 macrophages were treated with recombinant mouse IL-34 (50 ng/mL) for 72 h to observe the direct effects of IL-34 on unstimulated M0 macrophages. For M2 macrophage polarization experiments (M2 group), M0 macrophages were first incubated with 20 ng/mL IL-4 for 24 h to induce M2 polarization (identified by CD206^+^). Subsequently, the M2-polarized macrophages were treated with recombinant mouse IL-34 (50 ng/mL) for another 48 h to evaluate the regulatory effects of IL-34 on pre-induced M2 macrophages.

### 4.10. Cell Proliferation Assay

Macrophage proliferation was detected using the CCK-8 assay (Dojindo, Kumamoto, Japan). BMDMs were seeded into 96-well plates at a density of 5 × 10^3^ cells/well. After 24 h of culture, cells were treated with IL-34 or PBS for 24, 48, and 72 h. CCK-8 reagent (10 μL/well) was added to each well, and the plates were incubated at 37 °C for 2 h. The absorbance at 450 nm was measured using a microplate reader (Thermo Fisher Scientific).

### 4.11. Statistical Analysis

All data were expressed as mean ± standard deviation (SD). Statistical analysis was performed using GraphPad Prism 8.0 software (GraphPad Software, San Diego, CA, USA). Differences between two groups were compared using unpaired *t*-test, and differences among multiple groups were compared using one-way analysis of variance (ANOVA) followed by Tukey’s post hoc-test. *p* < 0.05 was considered statistically significant.

## Data Availability

The original contributions presented in this study are included in the article/Supplementary Materials. Further inquiries can be directed to the corresponding author.

## Supplementary Materials

The following supporting information can be downloaded at: https://www.mdpi.com/article/10.3390/ijms27156683/s1.

## References

[1] Saul D., Khosla S. (2022). Fracture Healing in the Setting of Endocrine Diseases, Aging, and Cellular Senescence. Endocr. Rev..

[2] Ai-Aql Z., Alagl A., Graves D., Gerstenfeld L., Einhorn T. (2008). Molecular mechanisms controlling bone formation during fracture healing and distraction osteogenesis. J. Dent. Res..

[3] Schlundt C., Fischer H., Bucher C.H., Rendenbach C., Duda G.N., Schmidt-Bleek K. (2021). The multifaceted roles of macrophages in bone regeneration: A story of polarization, activation and time. Acta Biomater..

[4] Guilliams M., Mildner A., Yona S. (2018). Developmental and Functional Heterogeneity of Monocytes. Immunity.

[5] Javaid M.K., Cinelli P., Lems W., Pierroz D.D., Sosa M., Åkesson K.E., Blank R.D., Brandi M.L., Chan D., Chandran M. (2024). Impact of osteoporosis and osteoporosis medications on fracture healing: A narrative review. Osteoporos. Int..

[6] Zou N.Y., Liu R., Huang M., Jiao Y.-R., Wei J., Jiang Y., He W.Z., Huang M., Xu Y.L., Liu L. (2024). Age-related secretion of grancalcin by macrophages induces skeletal stem/progenitor cell senescence during fracture healing. Bone Res..

[7] Ambrosi T.H., Marecic O., McArdle A., Sinha R., Gulati G.S., Tong X., Wang Y., Steininger H.M., Hoover M.Y., Koepke L.S. (2021). Aged skeletal stem cells generate an inflammatory degenerative niche. Nature.

[8] Pajarinen J., Lin T., Gibon E., Kohno Y., Maruyama M., Nathan K., Lu L., Yao Z., Goodman S.B. (2019). Mesenchymal stem cell-macrophage crosstalk and bone healing. Biomaterials.

[9] Lelios I., Cansever D., Utz S.G., Mildenberger W., Stifter S.A., Greter M. (2020). Emerging roles of IL-34 in health and disease. J. Exp. Med..

[10] Van Raemdonck K., Umar S., Palasiewicz K., Volin M.V., Elshabrawy H.A., Romay B., Tetali C., Ahmed A., Amin M.A., Zomorrodi R.K. (2021). Interleukin-34 Reprograms Glycolytic and Osteoclastic Rheumatoid Arthritis Macrophages via Syndecan 1 and Macrophage Colony-Stimulating Factor Receptor. Arthritis Rheumatol..

[11] Amarasekara D.S., Yun H., Kim S., Lee N., Kim H., Rho J. (2018). Regulation of Osteoclast Differentiation by Cytokine Networks. Immune Netw..

[12] Dang T.V.T., Mehdi A.M., Kumari S., Khosrotehrani K., Frazer I.H., Hume D.A., Chandra J. (2025). The Involvement of IL-34 in Inflammatory and Malignant Skin Diseases. J. Investig. Dermatol..

[13] Nian Z., Dou Y., Shen Y., Liu J., Du X., Jiang Y., Zhou Y., Fu B., Sun R., Zheng X. (2024). Interleukin-34-orchestrated tumor-associated macrophage reprogramming is required for tumor immune escape driven by p53 inactivation. Immunity.

[14] Nakajima S., Mimura K., Saito K., Min A.K.T., Endo E., Yamada L., Kase K., Yamauchi N., Matsumoto T., Nakano H. (2021). Neoadjuvant Chemotherapy Induces IL34 Signaling and Promotes Chemoresistance via Tumor-Associated Macrophage Polarization in Esophageal Squamous Cell Carcinoma. Mol. Cancer Res..

[15] Gargalionis A.N., Adamopoulos C., Vottis C.T., Papavassiliou A.G., Basdra E.K. (2024). Runx2 and Polycystins in Bone Mechanotransduction: Challenges for Therapeutic Opportunities. Int. J. Mol. Sci..

[16] Mognetti B., Marino S., Barberis A., Martin A.-S.B., Bala Y., Di Carlo F., Boivin G., Barbos M.P. (2011). Experimental stimulation of bone healing with teriparatide: Histomorphometric and microhardness analysis in a mouse model of closed fracture. Calcif. Tissue Int..

[17] Loi F., Córdova L.A., Pajarinen J., Lin T.H., Yao Z., Goodman S.B. (2016). Inflammation, fracture and bone repair. Bone.

[18] Zhang J., Akiyama K., Mun A.Y., Tagashira R., Zou T., Matsunaga N., Kohno T., Kuboki T. (2024). Age-Related Effects on MSC Immunomodulation, Macrophage Polarization, Apoptosis, and Bone Regeneration Correlate with IL-38 Expression. Int. J. Mol. Sci..

[19] Orecchioni M., Ghosheh Y., Pramod A.B., Ley K. (2019). Macrophage Polarization: Different Gene Signatures in M1(LPS+) vs. Classically and M2(LPS-) vs. Alternatively Activated Macrophages. Front. Immunol..

[20] Jin C., Liang J., Wu J., Han X., Zhou Y., Li B., Sun W., Su J., Sun J., Wan S. (2026). Temporal Immunomodulatory Hydrogel Regulating the Immune-Osteogenic Cascade for Infected Bone Defects Regeneration. Adv. Mater..

[21] Baghdadi M., Ishikawa K., Nakanishi S., Murata T., Umeyama Y., Kobayashi T., Kameda Y., Endo H., Wada H., Bogen B. (2019). A role for IL-34 in osteolytic disease of multiple myeloma. Blood Adv..

[22] Kalyanaraman H., China S.P., Cabriales J.A., Moininazeri J., Casteel D.E., Garcia J.J., Wong V.W., Chen A., Sah R.L., Boss G.R. (2023). Protein Kinase G2 Is Essential for Skeletal Homeostasis and Adaptation to Mechanical Loading in Male but Not Female Mice. J. Bone Min. Res..

[23] Kranz S., Heyder M., Rabe U., Liu P., Mrozinska A., Guellmar A., Berg A., Steen D., Tuckermann J., Watts D.C. (2023). Osseointegration of photodynamic active biomaterials for bone regeneration in an animal bone model over a period of 12 months. Dent. Mater..

[24] Xu J., Fu L., Bai J., Zhong H., Kuang Z., Zhou C., Hu B., Ni L., Ying L., Chen E. (2021). Low-dose IL-34 has no effect on osteoclastogenesis but promotes osteogenesis of hBMSCs partly via activation of the PI3K/AKT and ERK signaling pathways. Stem Cell Res. Ther..

[25] Ni B., Zhang D., Zhou H., Zheng M., Wang Z., Tao J., Han Z., Ju X., Tan R., Gu M. (2024). IL-34 attenuates acute T cell-mediated rejection following renal transplantation by upregulating M2 macrophages polarization. Heliyon.

[26] Du J., Liu Y., Wu X., Sun J., Shi J., Zhang H., Zheng A., Zhou M., Jiang X. (2023). BRD9-mediated chromatin remodeling suppresses osteoclastogenesis through negative feedback mechanism. Nat. Commun..

[27] Akhter N., Contreras J., Ansari M.A., Ducruet A.F., Hoda N., Ahmad A.S., Gangwani L.D., Bhatia K., Ahmad S. (2024). Remote Ischemic Post-Conditioning (RIC) Mediates Anti-Inflammatory Signaling via Myeloid AMPKα1 in Murine Traumatic Optic Neuropathy (TON). Int. J. Mol. Sci..

