# Supplementary material for: IL-34 Regulates Macrophage Polarization and Bone Defect Healing in Aged Mice
Source: Int J Mol Sci. 2026 Jul 27;27(15):6683. doi: 10.3390/ijms27156683 (PMC13466693; doi:10.3390/ijms27156683)
Supplement: Supplementary file 1 [file ijms-27-06683-s001.zip › ijms-4414171-supplementary.pdf]

*Supplementary Figure S1. Genetic knockout negative control for IL-34  antibody specificity.*

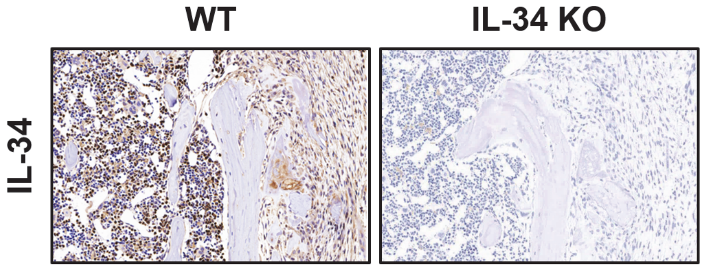


**Figure S1.** The paraffin sections were archived specimens from Cyagen KO mice. All IHC staining and photography were completed during revision by the first author; all images are original and unpublished. Specific staining only appeared in wild-type tissue, while no positive signals were detected in IL-34 KO sections, verifying antibody specificity.
